# Organizational structures, coordination, and financing mechanisms among governmental emergency medical teams in the European region: a cross-case analysis

**DOI:** 10.3389/fpubh.2026.1820843

**Published:** 2026-04-13

**Authors:** N. D. Zwanenburg, H. von Reding, J. von Schreeb, N. Amat Camacho

**Affiliations:** Global Public Health Department, Center for Research in Healthcare in Disasters, Karolinska Institutet, Huddinge, Sweden

**Keywords:** classified, coordination, emergency medical team, European, financing, governmental, organizational structure

## Abstract

**Introduction:**

Emergency Medical Teams (EMT) are groups of health professionals and support staff who provide direct medical care during disasters, outbreaks, and conflicts. Defining strong EMT organizational structures, coordination, and financing mechanisms is a crucial part of EMT development and is expected to impact the timeliness and effectiveness of EMT response. Yet there is a lack of research exploring these aspects. This study aimed to map and compare organizational structures, coordination, and financing mechanisms of classified governmental EMTs in the World Health Organization (WHO) European Region.

**Methods:**

Qualitative document analysis and cross-case comparisons were used. Eight classified governmental EMTs in the European region shared documentation: DEMA – DEMH EMT2 (Denmark), NOR EMT (Norway), EMT2 Toscana (Italy), UK-EMT, Swiss Humanitarian Aid, START—AECID (Spain), L-EMT (Lithuania), and RO. EMT1 Bucharest (Romania). Documents were analyzed using both deductive and inductive selective coding, and themes were compared across cases using a matrix.

**Results:**

While all adhering to the WHO EMT standards, EMTs exhibit considerable variation in organizational models. Some of the most notable differences are found in their organizational ownership and management, human resource management policies, deployment activation procedures, logistics arrangements, and EMT financial responsibility.

**Conclusion:**

The results of this study provide insight into how governmental EMTs can be structured, which will guide countries in future EMT development and reform. Building on the foundations offered in this study, future research should generate evidence about key organizational policies and their practical implications.

## 1 Introduction

Earthquakes, conflicts, disease outbreaks, and sudden-onset disasters are emergencies that can increase the demand for health services and overwhelm the resources and capabilities of healthcare systems. In such instances, Emergency Medical Teams (EMTs) can be deployed to support local capacities (1). EMTs are self-sufficient, trained, and equipped medical teams—managed by non-governmental organizations (NGOs), national, regional, and local governments, or the military—that provide medical care following emergencies.

The 2010 Haiti earthquake highlighted both the lifesaving potential and shortcomings of international medical responses. Nearly 300 teams were deployed (2) and saved many lives (3). However, evaluations by the Pan American Health Organization (3) concluded that many responders lacked adequate preparation, coordination, and competencies for the medical needs they faced and underscored the need for principles, quality criteria, and minimum standards for EMTs (1). The World Health Organization (WHO) EMT Initiative was launched in response to those needs, building on the first EMT technical guidelines published in 2013 (1), and were updated in 2021 (2). The guidelines (so-called “Blue Book”) establish a classification system for EMTs and define Core Standards to ensure quality of care. To be classified by the WHO for international deployment, EMTs must follow a standardized 8-step classification pathway (2).

The EMT Core Standards, divided into 96 Key Priority Areas (KPAs), define the overarching requirements for EMT operations (2). Clinical and technical standards are more prescriptive and designed to ensure that EMTs deliver a minimum standard of care. In contrast, the core standards addressing EMT features such as organizational leadership and financing are more generic, meaning they describe broad expectations to allow adaptation to different national or organizational contexts (2). This approach leads to a highly diverse global landscape of EMT organizational models (4).

The WHO EMT initiative encourages research and evidence generation as a means to strengthen knowledge and practice within the EMT community. These efforts are reflected in the growing body of literature on topics such as EMT deployment reviews (5–8), field hospital information systems (9, 10), team dynamics (11), or EMT training (12–14). Reviews on EMT management and funding from a global perspective have been conducted (4, 15). However, to the authors’ knowledge, no previous study has systematically analyzed the organizational structure and coordination mechanisms of governmental EMTs in the European context. Moreover, financing of EMTs is poorly documented, such as financial amounts required for operation and maintenance, specifically for governmental EMTs (4). The need for a review of EMT governance structures and the strengthening of research in this domain is highlighted under Strategic Objective 1 of the European EMT Regional Action Plan 2024–2030, aligned with the global EMT 2030 strategy (16).

This study aimed to map and compare organizational structures, coordination, and financing mechanisms of classified governmental EMTs in the WHO European Region. Through identifying similarities and variations in policies and procedures, this study illustrates the diversity of organizational models across governmental EMTs and provides an overview of EMT systems that can serve as a reference to governments and organizations globally seeking to establish EMTs.

## 2 Methodology

### 2.1 Study design

This study employed a qualitative document analysis, which is commonly used in political science research to enable impartial and consistent analysis of written policies (17). Document analysis was deemed the most appropriate method given that EMTs, as part of the WHO classification process, must develop a comprehensive package with documents detailing their organizational structures, coordination mechanisms, and financing mechanisms – normally in the form of Standard Operating Procedures (SOPs). The Blue Book was chosen as the framework guiding the study, selecting KPAs that define organizational structures, coordination, and financing mechanisms during stand-by (i.e., not during deployment).

Data was structured using a cross-case framework analysis, originally developed for large-scale social policy research, and has become increasingly common in medical and health research (18). Cross-case analysis is a systematic approach and facilitates structured comparison across multiple cases and themes through the use of matrix-based data charting (19).

### 2.2 Study setting

The WHO European region was selected as the study setting because it hosts the highest number of classified EMTs (30 EMTs classified at time of manuscript submission). This is about half of the classified EMTs globally, including governmental EMTs (2, 20).

### 2.3 Study population

The study population was defined by applying specific inclusion and exclusion criteria. Given substantial differences between non-governmental and governmental EMTs in terms of autonomy, resources, and political dynamics (15), only governmental EMTs were included. To fit the inclusion criteria, they had to be classified for international deployment by the WHO before September 2025. EMTs that were based in countries engaged in active conflict were excluded, considering potential challenges in accessing data.

The sample was compiled from the most recent available WHO classification list (20). In the WHO European Region, 17 government EMTs had been classified across 13 countries. After excluding EMTs from countries engaged in active conflict, 15 EMTs were contacted for participation in the study.

### 2.4 Data collection

EMT focal points were contacted via email with a list of requested documents and a two-page overview of the study’s aim and design. To support more targeted data collection, EMTs were asked to provide SOPs addressing the Core Standards for Administration and Organizational Management (KPA 1–6, 8), Human Resources (KPA 11, 13, 15, 17), and Coordination of EMTs (KPA 44, 45) (Table 1). Additionally, they were invited to share other documentation, such as organizational profiles or organigrams, leadership protocols, decision-making guidelines, activation workflows, deployment plans, financial policies, and funding arrangements, if not already included in the listed SOPs.

**Table 1:** Selected core standards and corresponding KPAs (2).

Title	KPA	Descriptor
**Administration and organizational management** Governments and organizations that deploy EMTs must have an administration and management system in place to ensure the policies, strategy and leadership of their organization is set up in such a way as to be able to form, finance and safely deploy an EMT within the time frames they have declared.		
Policies and documentation	1	Central repository of documents, policies and SOPs or an operational manual accessible to team members that cover aspects of the guiding principles, standards and technical SOPs applicable to their type and size.
Organizational leadership	2	Clear leadership structures to manage strategy and organizational stewardship and to decide on deployment in the shortest possible time frame during emergencies.
Finance and fundraising	3	Financial structures and governance in place for maintenance of EMT capacity in an ongoing state of readiness, as well as rapid access to funds to deploy within the time frames the team has declared.
	4	Fundraising in accordance with agreed ethical standards that does not inappropriately use patients or clinical images.
Risk management, safety, and security (at institutional level)	5	Strategies and systems to ensure deployments are preceded by planning and risk assessment, and that risk management, safety, and security policies and procedures are followed prior to and throughout EMT deployments.
Remote support for deployed teams	6	The ability to support teams while deployed, through headquarters or other forms of remote support, including the management of resupply of staff and consumables as required and withdrawal and evacuations.
External liaison, media and communications	8	Policies, procedures, and designated staff managing external liaison with relevant ministries, other organizations, and the media.
**Human resources** Human resources (HR) make up the most valuable part of any EMT. An HR management system must be in place for an EMT to be operational and to ensure appropriate duty of care to its members. In the phases of pre-deployment, EMTs must have policies and procedures on deployment and post-deployment regarding such matters as staff recruitment, health screening, and insurance. Team members should be asked to sign and respect a code of conduct. Policies on remuneration (or lack thereof), insurance (or equivalent), and indemnity cover for deployment should be clearly explained in the recruitment phase.		
**Pre-deployment preparedness**		
Recruitment, selection and health clearance	11	Comprehensive recruitment, selection and screening process for EMT members, including physical and mental health checks to ensure fitness and suitability to deploy.
Child protection checks	13	Policies and procedures to certify that local staff can be hired or contracted if required and screened for the purposes of risk protection.
Register database and roster management	15	Comprehensive HR management system that considers the correct numbers and types of staff required to deploy the team in a timely manner, and with adequate skill mix (both clinically and related to deployment experience).
	17	A plan to rotate staff to guarantee experience and mentorship is provided to the next generation of responders while ensuring to minimize impact on quality and safety of care.
**Coordination of EMTs** EMTs agree to monitor the national, and if relevant, regional and global situation during acute emergencies. They agree to accept initial impact assessments by relevant local authorities and to offer and/or respond to requests for assistance in a timely manner		
Activation mechanisms	44	National EMTs have mechanisms to be activated by national authorities through the health Emergency Operating Centre (EOC) or similar mechanism.
	45	National Disaster Management and Emergency Health Preparedness and Response Plans and SOPs include provisions for deploying national response teams such as EMTs.

Eight EMTs from the following countries submitted documentation: DEMA – DEMH EMT2 (Denmark), NOR-EMT (Norway), EMT2 Toscana (Italy), UK-EMT (United Kingdom), Swiss Humanitarian Aid (Switzerland), START – AECID (Spain), L-EMT (Lithuania), and RO. EMT1 Bucharest (Romania). See Table 2 for the full list of participating EMTs including classification according to EMT Typology.

**Table 2:** Participating EMTs.

Country	Team name	Type*	Referred to as**
Italy (Tuscany)	EMT2 Toscana	T 2	Tuscany EMT
Lithuania	LEMT	T 1 M	Lithuania(n) EMT
Romania	RO. EMT1 (Bucharest)	Romania is T 1 F	Romania(n) EMT
Denmark	DEMA—DEMH EMT2	T 2	Denmark/Danish EMT
Switzerland	Swiss Humanitarian Aid	SCT (RMNCH)	Switzerland/Swiss EMT
Norway	NOR EMT	Norway is T 1 F + M	Norway/Norwegian EMT
Spain	START—AECID	T 2 + T1 F	Spain/Spanish EMT
United Kingdom of Great Britain and Ireland	UK EMT	T 1 + SCT	UK EMT

^*^The Blue Book outlines the classification of EMTs by mobility and level of care provided, which allows harmonization of terminology and facilitates effective and rapid allocation of EMTs according to their capacities (2). Type 1 (T1) can be mobile (M) or fixed (F) and provide basic care and referrals, mainly focused on prehospital and outpatient care. Type 2 (T2) adds surgical and inpatient services. Type 3 (T3) adds complex referral and intensive care. Specialized Care Teams (SCT) deliver targeted services such as Reproductive, Maternal, Newborn and Child Health (RMNCH), mental health support, and outbreak response.

**For consistency, all EMTs are referred to using standardized English designations.

### 2.5 Data analysis

Qualitative document analysis involved the coding and extraction of relevant passages and organizing them into themes, categories, and case examples (21). As EMTs varied in the scope of materials submitted, a pre-selection of documentation was undertaken. Documents were screened for language, duplication, and relevance. Only English-language documents or those with translations provided by the EMTs were included. Where limited translations were required (e.g., budget-line descriptions of the Danish EMT), a secure translation tool was used without uploading full documents. Only materials directly relevant to the research questions were analyzed. Data was securely stored and not shared beyond the research team.

To guide the initial phase of data analysis, the selection of Core Standards and Key Priority Areas from the EMT guidelines (Table 1) that targeted data collection also served as a deductive framework for analysis. A coding scheme was developed by extracting keywords from the description of these standards, which guided the initial coding phase. To account for data outside this predefined coding scheme, inductive coding was also applied, resulting in a hybrid coding approach.

All documents underwent dual coding to enhance consistency and reliability. The iterative process allowed for refinement of codes and themes, ensuring that the analysis captured both conformity with EMT guidelines and emerging practices not explicitly addressed in the EMT standards. Coding was performed using qualitative data analysis software QDAcity, which facilitated clustering of codes and recognition of broader themes across the dataset. Coded data was systematically charted in a matrix, grouped into themes, and analyzed across cases to identify similarities and variations across EMTs.

As part of the validation process, the matrix and the narrative of the results were shared with all EMTs for review. Since the NOR EMT was undergoing policy revisions that were not yet reflected in the SOPs at the time of data extraction, these changes were incorporated following team feedback to ensure the dataset reflected the most up-to-date information.

### 2.6 Ethical approval and informed consent

This study was reviewed by the Swedish Ethics Review Authority (Etikprövningsmyndigheten) and was deemed exempt, as it does not involve interventions on research subjects or the processing of sensitive personal data. All EMT focal points were informed about the study objectives and procedures via an invitation email, and the provision of documentation was considered voluntary informed participation.

## 3 Results

The findings are organized around five thematic areas: EMT organizational structures, human resource (HR) management, deployment activation mechanisms and procedures, logistics, and financial mechanisms. Each thematic area is further divided into sub-themes to capture specific practices across the different themes. The results section provides a comparative overview of these themes, noting that the level of detail varies between EMTs depending on the depth of the available documentation. A matrix summarizing the themes discussed is provided in Annex 1 of the Supplementary material.

### 3.1 EMT organizational structures

EMTs are required to have clear leadership structures in place to manage strategic and organizational stewardship (KPA 2). The governmental EMTs are organized under a variety of governance models, ranging from centralized structures to more decentralized or hybrid arrangements involving regional authorities, private, or non-governmental partners.

Some EMTs are integrated within civil protection or emergency management agencies (Denmark, Tuscany, Romania, Lithuania), while others operate under foreign affairs or international development cooperation agencies (the UK, Spain, Switzerland), or under the Ministry of Health (Norway).

While most EMTs separate the ownership or oversight from the function of maintenance and coordination of the EMT, Denmark and Spain have both these functions under the same agency. All EMTs included in the study are managed at the national government level, except for the regional governmental team from Tuscany in Italy. The EMT2 Toscana has a decentralized management model in which both the National and Regional Departments of Civil Protection are involved.

In some EMTs, the management is (partly) delegated to NGOs (UK and Tuscany). Operational management of the Tuscany EMT is shared with the NGO Gruppo Chirurgia d’Urgenza (GCU) (Emergency Surgery Group). For the UK EMT, oversight is provided by the Foreign, Commonwealth, and Development Office (FCDO), and full operational maintenance has been delegated to the specialized NGO UK-Med. Notably, UK-Med is also classified as a non-governmental EMT.

### 3.2 Human resources management

#### 3.2.1 EMT staff roster and recruitment

EMTs are required to have a comprehensive recruitment, selection, and screening process for their members (KPA 11). EMT rosters consist of specialized medical, technical (e.g., WASH specialist), and logistics personnel. A multi-sourcing approach is most common, combining staff from public health systems, hospitals, private contractors, and civil protection structures.

All EMTs recruit trained medical staff from the national public health systems, mostly hospitals. Most EMTs recruit logistics staff from civil protection structures (Spain, Tuscany, Denmark, and Norway), such as fire and rescue departments (Romania and Lithuania). The Swiss team recruits from the Humanitarian Aid Division. In Tuscany, a column of partner organizations makes up the regional civil protection system, and the EMT draws logistics personnel from all these organizations. Spain recruits the technical coordination personnel through an external contractor. Norway specifies that it draws technical personnel such as public health experts from the Norwegian Institute of Public Health.

The UK EMT draws from the UK-Med register, which combines a core group of deployable staff including international staff and professionals recruited from the National Health Service. Since UK-Med is involved in both the governmental and non-governmental EMT, there is a shared staff pool. The UK EMT stands out with detailed HR SOPs describing policies and procedures not documented by other EMTs. The ‘Workforce Planning Cycle’ is an ongoing activity to analyze current and future workforce needs, identify gaps, and address shortages on an ongoing basis. Additional policies include a peak demand workforce scenario and an assessment of ratios needed for each role. All EMTs maintain a minimum 1:5 ratio for each position, in line with the WHO requirements. The UK EMT applies a 1:6 ratio, with some roles maintained at a ratio of 1:8 or 1:10 based on peak demand scenarios. Since the UK EMT also deploys longer term, there is a rest and recuperation policy in place.

Most EMTs describe formal nomination or structured application procedures. Some rely on internal nomination and assessment procedures within government agencies (Denmark, Norway), while others use open or continuous recruitment coordinated by HR departments or partner organizations (Switzerland, UK, Tuscany, Lithuania, Spain). In Romania, recruitment takes place annually, with major hospitals and fire and rescue units nominating staff for the EMT roster.

#### 3.2.2 EMT roster management system

Roster management systems differ across EMTs. Denmark and Tuscany use dedicated software that allows them to keep staff profiles up to date and support rapid deployments. Norway and Lithuania maintain centralized databases within national authorities to guide data management and staff selection. Tuscany, Spain, and Romania have separate rosters between agencies.

#### 3.2.3 Employer release mechanisms and contracting

Employer release mechanisms—e.g., agreements with institutions to rapidly release staff for deployments—were reported by several EMTs, ranging from formalized agreements and prearranged on-call systems to more reactive, *ad hoc* approaches. Denmark, Norway, Romania and Switzerland have agreements that enable hospital staff to be released for international missions. In Norway, hospitals are committed to supporting the EMT but may refuse release if doing so would significantly affect their own operations and preparedness. The Department of Emergency Situations in Romania has legal coordination over the institutions releasing medical and logistics staff, and therefore release is prearranged. Lithuania and Tuscany describe more *ad hoc* approaches such as negotiating release in case of deployment.

The UK describes one of the most formalized systems: agreements between UKMed, the UK EMT, and the National Health Service allow staff to commit in advance to on-call periods of up to 2 months per year, during which employers guarantee release for rapid overseas deployment within 12–24 h for missions of about 3 weeks. Staff who are not on the register through the National Health Service release mechanism can generally be available for longer periods of at least 6 weeks.

Staff are either employed by the organization that has ownership of the EMT (Denmark, Norway, Switzerland), provide options for short-term contracts (UK), or remain under the existing contracts with their institutions of origin (Spain, UK).

### 3.3 Deployment activation mechanisms and procedures

EMTs generally monitor global alert and early warning systems as part of their preparedness activities. When a sudden-onset disaster occurs, EMTs initiate preparedness and pre-deployment actions immediately, often before a formal request for international assistance is issued. EMTs follow standardized deployment activation mechanisms and procedures to ensure rapid and effective deployment, which are described in this chapter. While these activities are standardized in principle, their implementation varies between EMTs. All EMTs await formal acceptance of Emergency Medical Team Coordination Cell (EMTCC) and the affected country before deployment.

#### 3.3.1 Deployment decision-making

EMTs are required to have clear leadership structures in place that enable deployment decisions to be made as quickly as possible during emergencies (KPA 2).

Most EMTs have visualized their decision-making processes in flow-charts, including the stakeholders involved or consulted at each stage. In Norway and Denmark, multiple ministries are consulted in the decision-making process. In Spain, the director of the Agency for International Development Cooperation decides after consultation with the Humanitarian Action Directorate and sub-directorates. The Swiss EMT places decision-making authority with the head of the Humanitarian Aid Unit, who oversees its operations, logistics, and expert roster. For the Tuscany EMT, a specific process takes place. The National Focal Point forwards a request for assistance to the Regional Health Referent, who is in charge of convening a committee including delegates from the regional health department, regional civil protection department, and the president of the NGO Emergency Surgical Group. This committee consults and collectively makes the deployment decision.

In Romania and Lithuania, deployment requires formal approval from the Prime Minister. In Romania, the Department of Emergency Situations coordinates the process and consults with the Minister of Interior, but the Prime Minister’s consent is decisive. The UK EMT differs from all others: final authority rests with the Operations Director of the NGO UK-Med, in consultation with the Foreign Affairs office.

Decision timelines were only indicated by Denmark and Tuscany. Denmark approves deployment within 6–8 h after an international incident, and Tuscany’s Steering Committee decides within about 5 h of a request.

#### 3.3.2 Coordination team

Several EMTs (Denmark, Norway, the UK, and Tuscany) report the activation of a dedicated team or cell that is rapidly mobilized when deployment is likely. These teams coordinate and delegate activation procedures, such as staff mobilization, team configuration, liaising with the EMTCC, the host country, and activating all necessary resources to ensure rapid deployment. The activation of these “coordination teams” can be triggered by either the Director General of the respective agency (Denmark and Norway), the Regional Health Referent (Tuscany), or the Operations Director (UK).

In Denmark, an operational cell of 10–15 staff can be mobilized within an hour, drawing personnel from the Emergency Management Agency’s logistics, medical, communications, and HR departments. Norway activates an “HQ Staff Team” with similar functions. The Tuscany EMT activation is overseen by the coordinating committee, supported by a secretariat, and after deployment is indicated, a Support Team is activated. Roles in this support team not described by Denmark and Norway include an insurance officer, a documents officer, and an information technology officer. The UK EMT relies on a Project Team that includes representatives from operations, logistics, HR, finance, communications, and training functions.

#### 3.3.3 Staff activation and mobilization

The policies of staff activation and mobilization are not described under a specific KPA, and EMTs described policies that fit their decision-making procedures. Some EMTs start to mobilize or send out pre-alerts to their roster if there is an indication for deployment. Therefore, there can be multiple (pre)alerts being sent out as the emergency evolves, involving updates on the possible deployment. Teams rely on SMS, phone calls, email, automated alerts, or WhatsApp and Telegram groups to notify members and confirm availability.

Most EMTs expect fast responses and readiness from their personnel. Personnel are generally required to confirm availability within a short timeframe, ranging from 2 hours in Romania to 24 h in Spain and Switzerland. Norway, Spain, and Switzerland expect members to maintain personal preparedness—such as arranging transport options and following packing guidelines tailored to mission environments.

#### 3.3.4 Team configuration

EMTs should have a system that considers the correct number and types of staff required to deploy the team in a timely manner, and with an adequate skill mix (KPA 15). While this KPA does not prescribe specific procedures and policies, all EMTs describe structured processes for configuring teams. While basic principles are similar across countries, the policies differ in structure and detail, and in the case of the UK EMT, additional policies are documented.

All EMTs generally start with a long-list of available staff generated through HR systems, narrowing down to a selection of the staff roster for the actual deployment. Spain describes a case-by-case team configuration process. The UK team configuration process is a systematized three-phase process: defining operational objectives, planning team composition, and final mobilization. Responsibility in the different phases is with Operations (Phase 1), HR (Phase 2), and Technical Support (Phase 3). The UK team also applies a “no-regrets” policy for team configuration, meaning that multiple candidates can be prepared for each role without guaranteeing deployment. Romania applies a similar no-regrets policy.

Two EMTs describe diversity considerations (UK and Denmark). The UK describes the teams as strongest when they include a mix of people, considering personality types and traits, gender, and ethnicity alongside technical qualifications.

#### 3.3.5 Staff rotation and roster development

EMTs are required to have a plan for staff rotation that ensures experience and mentorship are passed on to the next generation of responders (KPA 17). However, roster development and staff rotation to ensure training and experience is passed on is not described in a standardized manner across EMTs.

The Danish EMT describes the planning for staff rotation in the cycle of a deployment, since different specialties are required at different stages of deployment. Prioritizing technical specialists and logisticians during the building and dismantling of the field hospital and adapting medical expertise as needs evolve in the field. Arrival and deployment timelines for each specialty are specified in a timeline for a standard deployment. Spain describes a standard 15-day maximum rotation for the roster by default.

Denmark emphasizes competency development, Tuscany specifies mentoring, and the UK refers to register development since each deployment provides an opportunity to improve skills and strengthen future responses. The Swiss EMT aims to balance senior and junior experts, although they describe that this is challenged by generational change and shortages of humanitarian experience compared to medical expertise.

#### 3.3.6 Assessment team deployment

Deploying an assessment team—ahead of the full EMT module – is not specified as a minimum standard; however, several EMTs describe this in their policies. The Danish EMT describes this element in detail and outlines a team of 8 members with individuals who are experts in their fields with minimum the Team Leader and a medical doctor, who bring accommodation equipment, medicine, food, and cash to ensure 72 h of self-sufficiency. The Tuscany EMT deploys civil protection officers from the national and regional departments, an experienced logistician, and an experienced healthcare provider. The Norwegian EMT specifies a minimum composition of an EMT Team Leader, and a Senior Medical Officer and Spain describes a 2-person team. The Swiss EMT deploys Rapid Response Teams of minimum 3 members that can scale up to 30 members.

### 3.4 Logistics

#### 3.4.1 Logistical partners

For several EMTs, the military plays a direct role in logistics operations, either by managing warehouses or providing transport support such as loading aircraft and coordinating shipments (Denmark, Spain, Switzerland). Other EMTs maintain centralized storage managed by civil protection or emergency services (Denmark, Norway, Lithuania, Romania). Tuscany receives support from the regional civil protection system, including multiple partner organizations such as the Local Committee of the Red Cross. Norway receives support from a commercial logistics partner that provides transport and supply chain support.

#### 3.4.2 Pharmacy and medical equipment storage

Romania, Tuscany, and Norway have agreements in place to store prearranged batches of medicines at hospital pharmacies for rapid activation of resources. Rotation of soon-to-expire drugs is the responsibility of the pharmacy. Norway and Tuscany specifically state that soon to expire drugs are rotated in the hospital stock to reduce waste. Spanish team has access to medical supplies through a pharmacy contractor that supplies when needed. Tuscany reports that equipment requiring continuous operation, such as laboratory devices, remains in hospital use and is made available at deployment.

Denmark and Lithuania store medications and medical equipment at ‘state’ warehouses. Lithuania specifies that their maintenance and renewal are ensured through existing mechanisms. Denmark maintains stock for approximately one week of operations in its own warehouse, with additional consumables supplied through a national warehouse. Medicines are delivered within 6–12 h of a request, and hospitals can provide up to 30 units of blood.

### 3.5 Financial mechanisms

#### 3.5.1 Funding responsibility

EMTs are required to have financial structures and governance in place to ensure the maintenance of the EMT capacity and an ongoing state of readiness, as well as rapid access to funds to deploy within the time frames the team has declared (KPA 3).

Funding responsibility generally lies with state ministries or government agencies, most often the ministries of foreign affairs or development cooperation agencies (Denmark, the UK, Spain, and Switzerland). In Lithuania funding is provided by the government through state budgets, as well as by the Ministry of Health. In Romania, financing is managed through national crisis management centers. Similar to Tuscany, where the EMT funding responsibility lies with the national and regional Department of Civil Protection and the NGO. In Norway, the funding responsibility depends on the requester of the EMT, so it could be that several ministries share funding responsibility or it is shared with the requester.

#### 3.5.2 Funding sources, access, and conditions

EMTs apply diverse funding models that combine public, NGO, and *ad hoc* sources. The Tuscany EMT exemplifies a mixed model, with member contributions and NGO-generated income (training, donations, reimbursements, and service provision). During missions, costs are covered jointly by the NGO and the Department of Civil Protection, which provides additional funds as needed. The Romanian EMT relies on state budget allocations for operations, training, and upgrades, while medical supplies are financed separately through a national hospital. To enable rapid financial support in areas where state funding may be bureaucratic (e.g., training, travel), the EMT remains a strong partnership with the SMURD Foundation, a well-known Romanian NGO designated to support the national emergency medical rescue system. The Swiss EMT operates under the International Cooperation Strategy and is funded through the budget of the Swiss Agency for Development Cooperation, approved by parliament.

The UK EMT combines core program funding with mission-specific grants managed by UK-Med. A contingency budget introduced in 2016 supports faster decision-making for smaller missions.

The Danish EMT can access standby funding pools (1.4 million USD total) through the Ministries of Foreign Affairs and Defense and allows initial funding access of 100,000 USD under a “no-regret” policy, before the final deployment decision is made to start the mobilization of resources. Both Norway and Denmark link funding eligibility with their membership of the Organization for Economic Co-operation and Development (OECD) Development Assistance Committee (DAC). Funding for deployment to DAC-eligible countries qualifies as Official Development Assistance (ODA) and is approved more quickly, while alternative arrangements may require alternative funding sources.

#### 3.5.3 EMT budget estimations

Only Denmark provided detailed information on EMT budget estimations. The Danish EMT provides a detailed budget for a three-week deployment, including a separate budget for the deployment of an assessment team. A 10% contingency is applied to personnel-related costs and a fixed 20% contingency to mission-specific costs, bringing the total estimated cost to approximately €2.7 million. Additionally, the budget lines specify which costs are eligible for EU Civil Protection Mechanism co-financing (covering up to 75% for transport and operational costs). The assessment team budget is approximately €74.000,00 and is not covered by the EU mechanism.

The UK EMT did not provide budget information; however, annual financial reports of the funding by the UK Foreign, Commonwealth and Development Office (FCDO) to UKMed provide insight into yearly ‘operational costs’ or the so-called ‘contingency budget’ (e.g., £4.68 million in 2023 and £7.14 million in 2024) of UKMed, with additional funding for specific missions (e.g., Gaza £2.75 M in 2024).

## 4 Discussion

This paper provides an overview of organizational structures, coordination mechanisms, and financing mechanisms of governmental EMTs in the WHO European Region. The eight EMTs included in this study have all been classified and adhere to the same EMT Core Standards described in the EMT guidelines. Despite this uniform classification, the themes presented provide significant variation in approaches to organizational structures, human resources policies, deployment activation procedures, logistics mechanisms, and financing arrangements.

The governmental EMTs are hosted by civil protection or emergency services agencies, or by the ministries of foreign affairs, international development, or health. Moreover, the EMTs operate under governance models that range from centralized systems with one or more national-level agencies to more decentralized arrangements that combine national and regional government involvement and NGO involvement. Regarding HR procedures, EMTs present different examples of staff recruitment pathways, employer release mechanisms, and roster management systems that are shaped by national systems and institutional partnerships. Each EMT describes similar procedures and activities taking place during activation; however, the implementation of deployment decision-making, staff activation and mobilization, the configuration of teams, and whether assessment teams are deployed differ across EMTs. Moreover, differences are evident in logistics arrangements, which are influenced by the nature of public and private partnerships. Although all EMTs must ensure readiness and rapid access to funding, descriptions of funding policies were limited in the documentation, which impeded a comparison. While the agency that has financial responsibility is generally outlined, details about how funds are accessed and the conditions attached to funding provision were not specified in the SOPs provided by the EMTs, with the Danish EMT being a notable exception in their transparency on funding policies.

While the results section is structured around thematic areas, which emphasizes variation across these domains, a different perspective emerges when examining the matrix (see Supplementary material) by EMT. Analyzing individual EMT cases reveals greater internal consistency, suggesting that organizational choices are more coherent than they appear when considered by theme alone. For example, in Denmark, the placement of the EMT within the Danish Emergency Management Agency shapes multiple thematic areas, including the organization of logistics personnel, decision-making authority during activation, agreements with military logistical partners, and the storage and management of pharmaceuticals and medical equipment. This underscores the value of presenting the data in matrix form, as it allows both cross-country comparison by thematic area and case-based analysis, making the interconnections between thematic areas more visible.

This study’s results provide similarities with a review of funding and management structures of EMTs outside of the European context, including Australia, China, India, Israel, Malaysia, and the Caribbean, blending NGO, military, and non-classified EMTs (4). Similar organizational structures were observed, such as EMT hosted by civil protection systems in Australia and China (4). China hosts five regional governmental EMTs (20), recruiting from fire and rescue departments (4). While coordination is mostly municipal, overall leadership is provided by the Chinese Ministry of Emergency Management (4), similar to the regional Tuscany example in Italy. Due to the exclusion of EMTs from countries engaging in active conflict, no military EMTs were included in our analysis, and no parallelism can be drawn with India and Israel in that respect. While the gap in literature on EMT organizational models impedes the comparison organizational models outside the European context, we can explore the potential implications of different organizational models and features, and how the findings of this study may be interpreted.

The diversity of organizational models of governmental EMTs appear closely tied to which institution(s) host(s) the EMT. The integration of an EMT into the existing structures of the national health department was identified as one of the four major challenges during the development of a national EMT in Papua New Guinea (22). In this case, it was observed that both the public health division and the medical standards division would be appropriate to host the EMT, since EMT services include both clinical and public health components. Also, the placement under one of these subdivisions would have important implications for the integration process, activation of pathways, and funding (22). Different institutions hosting the EMT come with different legal and operational environments that shape EMT organizational structures and mechanisms. In our comparative analysis, this observation is reflected in diversity of final authority on deployment decision-making ranging from a committee of institutional representatives deciding collectively, to the Prime Minister deciding on deployment. Variety was also observed in financial responsibility and employee contracting, for example.

While this study focused on governmental EMTs, the UK-EMT is an example of a hybrid EMT, providing the opportunity to deploy as an independent NGO or as a governmental EMT part of the UK’s official humanitarian assistance. EMT deployment decisions can be highly emotional and inherently political processes. Institutional interests, visibility, and media dynamics can shape both public perception and governmental decision-making regarding deployments (15). While literature on hybrid EMT models does not exist, the case of the UK EMT illustrates how an organizational set-up can provide flexibility in regard to the difficulty of deploying a governmental EMT in politically sensitive environments, such as conflicts.

While EMTs shared comprehensive documentation packages, actual insight into EMT costs remained limited and this is consistent with previous research indicating that few EMTs report financial data and policies (4, 23). The importance of clear and predictable financing structures is well established in the literature, as EMT deployments are costly (23). A distinction can be made between baseline costs (training, equipment, personnel) and response costs incurred during actual deployments (e.g., transportation, field operations). Challenges to calculate baseline costs for governmental EMTs can appear when expenses are covered by broader ministry or agency budgets without EMT-specific cost tracking. At the same time, response costs are challenging to estimate due to the unpredictable nature of EMT deployments. Different organizational choices, such as staff pool arrangements, logistics managed internally versus through private contractors, or transport provided by military versus civilian assets, likely have direct cost implications that remain undocumented. Greater clarity on EMT funding mechanisms would not only enhance accountability and EMT cost-effectiveness assessments, but also support improved preparedness, enabling better identification of financial gaps (23).

Funding and logistical constraints have been highlighted as continental challenges by the WHO Africa region (24), and similar concerns have also been reported in the Chinese context (25). Therefore, the European context of this study should be taken into consideration, specifically related to available funding for countries and EMTs to develop and deploy an EMT as part of the European Medical Corps with European Union financial support (26). Moreover, the Danish EMT budget included in this study, reflecting a high-income country context, is unlikely to be representative of the funding available to other EMTs within the region or beyond Europe.

Despite a clear methodology for requesting data, several EMTs contacted could not share documentation. Two EMT focal points noted that institutional restrictions prevented them from doing so. While challenges in accessing operational and policy documentation from EMTs persist (4, 15), knowledge exchange within the EMT network is gradually improving through the implementation of digital platforms and coordinated reporting mechanisms, in line with the EMT strategy. Partnership among EMTs is also showing a promising impact. A good example is the collaboration between the UK EMT and the Ethiopian EMT, through which best practices were shared regarding HR recruitment and training, including the implementation of web-based HR and training platforms that enabled cost-effective, accessible, and manageable learning solutions (26). Expanding knowledge exchange between EMTs should be strongly promoted as it contributes to mutual learning, improvement, and accountability.

### 4.1 Limitations

Several limitations should be considered. The analysis relied on documentation made available by the EMTs, in which certain procedures might not be described or not described in depth, even though they might be applied in reality. Some documents were originally in the national language and translated by the EMT, introducing a potential risk of minor inaccuracies, which were addressed through validation. In addition, the studied dimensions were documented by the EMT in varying formats and levels of detail, limiting the ability to compare across cases.

Given that the findings are highly context-specific, their transferability is inherently dependent on contextual factors. This study only included eight governmental EMTs in the WHO European Region. Further inclusion of the remaining governmental and non-governmental EMTs would likely reveal an even broader range of organizational structures and policies in the region. This study should therefore be regarded as an initial attempt to provide a systematic analysis and overview of the diversity of EMT systems that exist.

### 4.2 Future research recommendations

The themes identified in this study provide a useful foundation for future research examining EMT organizational structures across governmental, military-hosted, and NGO-led EMTs, as well as in other global regions. Applying these themes in subsequent work could support more systematic documentation of approaches to establishing EMTs across diverse contexts.

While this document-based approach allowed for structured and comparative analysis, it remains limited in capturing the practical realities of documented SOPs. Future research should integrate empirical data, such as interviews or field observations, to examine how policies and procedures are applied in real-world deployments. Those may include perceptions of intended versus the actual implementation of the described policies and how those affect timeliness and effectiveness of deployment.

Several features identified and discussed in this paper could be studied in more detail, including how the legal and operational environments of host institutions shape EMT development; the political dynamics of EMT deployment decision-making in general; and hybrid governmental–NGO EMT models and their potential benefits in complex settings. Further research could also explore human resources systems, particularly challenges related to recruiting, releasing, and retaining experienced staff and sustainable financing arrangements.

## 5 Conclusion

This study provides a first overview and comparison of documented organizational structures, coordination, and financing mechanisms of governmental EMTs in the WHO European Region, filling a critical gap in evidence-based guidance for EMT development. It identifies key elements that shape deployment readiness and offers a foundation for further research into how these organizational choices influence emergency preparedness and response in practice.

Although all EMTs adhere to the same standards, we found substantial variations in governance, public and private partnerships, decision-making authority, mechanisms for activation and coordination, and funding structures. These findings demonstrate that there is no single model for designing an EMT capacity. Instead, countries can build their EMT structures based on their national context and priorities while meeting WHO classification standards for international deployment. This study provides examples of the variety of EMT organizational choices, which is particularly relevant for governments currently establishing EMTs or seeking to reform their existing systems.

## Data Availability

The datasets presented in this article are not readily available because not applicable. Requests to access the datasets should be directed to nieves.amat.camacho.
