# Supplementary material for: Organizational structures, coordination, and financing mechanisms among governmental emergency medical teams in the European region: a cross-case analysis
Source: Front Public Health. 2026 Apr 13;14:1820843. doi: 10.3389/fpubh.2026.1820843 (PMC13111451; doi:10.3389/fpubh.2026.1820843)
Supplement: Supplementary file 1 [file Table_1.docx]

**Annex 1. Overview of documented** **EMT organizational structures, coordination, and financing mechanisms among governmental EMTs in the European region.**

| **EMT Name** | **DEMA - DEMH EMT2** | **EMT2 Toscana** | **Norwegian (NOR) EMT** | **UK-EMT** | **START - AECID** | **Swiss Humanitarian Aid** | **LEMT** | **RO. EMT1 (Bucharest)** |
| --- | --- | --- | --- | --- | --- | --- | --- | --- |
| **Country** | **Denmark** | **Italy (Tuscany)** | **Norway** | **The UK** | **Spain** | **Switzerland** | **Lithuania** | **Romania** |
| **3.1 EMT organizational structures** | | | | | | | | |
| **EMT ownership or oversight** | Danish Emergency Management Agency (DEMA) a governmental agency under the Ministry of Resilience and Preparedness | National Department of Civil Protection  (Mission control and international focal point)  The Regional Department of Civil Protection is in charge of the EMT.  Legal ownership is with the NGO *Gruppo Chirurgia d’Urgenza* (Emergency Surgery Group) | The Ministry of Health (MoH) and the Ministry of Justice (MoJ) share responsibility for NOR EMT, but the team is under the authority of MoH. | UK Foreign, Commonwealth and Development Office (FCDO) | Spanish Agency for International Development Cooperation (AECID) | Swiss Agency for Development Cooperation (SDC) | National Emergency Medical Services (NEMS) and Ministry of Health of Lithuania | General Inspectorate for Emergency Situations (GIES) |
| **EMT maintenance and operational management** |  | Regional Department of Civil Protection manages the EMT together with the Emergency Surgery Group | Directorate of Health (under the MoH) and the Directorate of Civil Protection (under the MoJ) | UK-Med, an NGO specialized in emergency medical care |  | Swiss Humanitarian Aid Unit an agency under the SDC | NEMS | The Department of Emergency Situations (DES), part of Ministry of Interior responding to the Prime Minister |
| **3.2 Human Resources Management** | | | | | | | | |
| **EMT staff roster and recruitment** | Medical staff come from hospitals within the national health system | Medical staff register through the NGO. The healthcare providers are volunteers of the NGO but employed in the National Health Service both from Tuscany and other regions | Medical staff: recruited through formal nomination via hospitals | Medical staff are recruited from hospitals and the National Health Service and apply to the UKMed volunteer roster  UKMed combines core deployable staff with a volunteer roster (including international staff) | Medical staff is a volunteer roster recruited from Spanish public health systems from all Spanish regions through open calls managed by AECID | Swiss Humanitarian Aid Unit has a roster of 550 humanitarian experts with 87 in the health expert group  Continuous recruitment via HR and partner organizations | Medical staff are recruited via hospitals across Lithuania  Uses open/continuous recruitment through HR or partner organizations | Medical staff are nominated once a year by (state) hospitals |
|  | Logistics staff come from DEMA through formal nomination and assessment | Logistics staff comes from a column of organizations that make up the region’s civil protection system | Team leaders/technical/logistics staff are recruited via Directorate of Civil Protection  Public health experts are recruited via the Norwegian Institute of Public Health |  | Technical team (technical coordination for medical, logistics and WASH aspects) are recruited via an external public contractor (AECID)  Logistics & WASH staff; volunteer roster; recruited from regional and local administration though agreements with fire departments and emergency medical services, and the NGO CESAL (kitchen) |  | Logistics staff come from fire and rescue departments | Logistics, and technical staff are nominated once a year by fire/rescue units (firefighter units are military) |
| **EMT roster management system** | Dedicated software (Everbridge) for maintaining profiles and supporting deployments. | The Emergency Surgical Group manages HR through dedicated software.  The logistics staff from the partner organizations are registered with the NGO, but documentation is handled by their respective organizations. | Maintains separate electronic personnel files with the Directorate of Civil Protection |  | Separate rosters for clinical and logistics staff; Excel-based system managed by AECID and institutional records. |  | NEMS maintains a centralized database containing individual staff files.  The Fire and Rescue Department maintains a database with individual staff files for all LEMT members assigned by Fire and Rescue. |  |
| **Employer release mechanisms and contracting** | Volunteer health personnel employed at national hospitals are able to utilize a national agreement allowing them to be released from duty in service of international organizations that the country collaborates with.  During deployment, DEMA employs and remunerates the volunteers. | The healthcare providers are volunteers of the Emergency Surgery Group but employed in the National Health Service (from Tuscany and other regions)  The employer is contacted to ensure departure clearance. | Formal release agreements are in place; however, hospitals may refuse if it affects their own operations and preparedness.  Both health care and technical personnel will be employed by the Directorate of Civil Protection during training and deployment. | National Health Service (NHS) agreements via UK-Med allow staff to on-call periods of up to 2 months/year with guaranteed release (12–24h notice) for 3-week missions. Non-NHS staff are available for longer deployments (6+ weeks).  Volunteers join the surge of responses temporarily, either on short-term contracts or under release agreements with their employers. | The staff deployed to missions will develop their work under the existing contracts with their institutions of origin. | The SDC has agreements with several of the larger hospitals regarding the quick and uncomplicated hiring of medical experts for rapid response missions. | Staff must notify their employers when participating in EMT activities. | The DES has legal coordination over the institutions (hospitals/fire unit) releasing staff, and therefore pre-arranged release is ensured.  Staff remain under existing contracts with employers and will receive additional daily allowance (35 euros). |
| **3.3 Deployment activation mechanisms and procedures** | | | | | | | | |
| **Deployment decision-making authority** | The Director General of DEMA | Steering committee  (regional health rep., civil protection rep., NGO president) | MoH and MoJ | Operations Director of UK-Med | Director of AECID | Swiss Humanitarian Aid Unit | The Prime Minister | The Prime Minister |
| **Decision-making process** | DEMA investigates the possibility of providing the EMT, if this is likely it activates the operations cell.  Multiple ministries consulted (Foreign Affairs and Defense). Deployment approved within 6–8 hours after an international incident.  Funding is approved within 12 hours. | The national focal point activates the regional health representative.  The Regional Health rep convenes the Steering Committee and activates the Operations Secretariat, and they evaluate the activation request, the minimum feasibility conditions for deployment.  The Steering Committee decides on deployment within 5 hours after requesting. | Directorate of Civil Protection and Directorate of Health make an initial assessment of the request and alerts relevant actors for decision making.  MoH and MoJ will always be involved in decision-making, but depending on the requesting party, other ministries, particularly the MFA may contribute to the decision-making process.  Hospitals decide on staff release | The Operations Director will convene a meeting with the FCDO to discuss deployment. The Operations Director will inform the Operations Lead as soon as a decision is made and/or whilst discussions are taking place.  In the high likelihood of deployment, the Operations Lead will move immediately to the deployment planning phase. | Decision made by Director of AECID after consultation with the Humanitarian Action Directorate and sub-directorates. | The Swiss Humanitarian Aid Unit is structured to enable very rapid decision-making and deployment.  Head of the Humanitarian Aid Division leads divisions of H-Operations / Rapid Response, Logistics, and Human Resources and therefore centralize decision making authority without approval of the without approval from the Swiss federal council. | Following a formal declaration by the Prime Minister of the Government of Lithuania, the EMT is activated. | The head of DES coordinates and consults with Minister of Interior and Prime Minister to receive formal approval. |
| **Coordination team** | Dedicated operational cell (10–15 staff)  Mobilizes within 1 hour of request  Includes logistics, medical, communications, and HR  Activated by the Director General | Steering Committee  Supported by secretariat and Support Team  Support team: Logistics, HR, Healthcare equipment, food, insurance, documents, finance, and IT officers.  Activated by Regional Health referent | HQ Staff team  Similar composition and function to Denmark’s operational cell  Activated by the Director General of the Directorate of Health and the Director General of the Directorate of Civil Protection | Project Team  Includes operations, health, logistics, HR, finance, communications, ravel coordinator, Supply chain advisor, training lead and warehouse manager.  Activated by Operations Director | AECID HQ Team |  | Crisis Cell  Activated at national level |  |
| **Staff activation and mobilization** | Standardized text or voice messages with detailed pre-deployment information as soon as there is reasonable certainty about deployment.  The candidates can respond via text or call whether they are available and or interested in a given deployment and position.  Staff confirm availability rapidly | Standardized text or voice messages with detailed pre-deployment information.  A pre-alert is sent to all roster members and the logistic support personnel.  Staff confirm availability through a quick answer app survey. | Mobilization through SMS, phone, or automated alerts.  Members maintain readiness (transport, packing). | Uses automated alerts (SMS, calls).  Staff confirm quickly. | Uses WhatsApp (directorates), Telegram (roster staff), phone calls, and emails. Availability confirmed within 24 hours. Members maintain personal preparedness (transport, packing). | Members receive initial calls with basic information from HR or the Field (place of deployment, time of deployment function, etc.).  Respond within 24 hours  Perform personal preparation for the mission | Pre-alert when Crisis Cell is activated, official SMS after government approval.  The team members negotiate availability for the mission with their respective employers.  Team members report back to the NEMS and declare availability for deployment. | Personnel receive pre-alert by phone/SMS followed by an alert to activate.  After the alert to activate message has been received, the personnel reports on duty to the unit within 90 minutes (as maximum for logistics staff) and 120 minutes (as maximum for medical staff).  . |
| **Team configuration and staff rotation** | The roster management system generates a list of candidates that responded, and the HQ selects and combines the team based on operational requirements, including gender composition, diversity, and competency development.  Staff rotation planned according to deployment cycle — technical/logistics staff prioritized for set-up and dismantling; medical staff adapted as needs evolve. | A list of available staff is reviewed using the databases of the Emergency Surgical group and the Regional Civil Protection database to verify that all requisites are met.  Mentoring emphasized as part of staff rotation and learning.  At least two shifts are selected for staff rotation. | The Directorate of Civil Protection and the Directorate of Health identify relevant team members in accordance with the request and initiate final alert for the selected team and deployment.  Rotation emphasizes the transfer of experience and competency development. | Three-phase process: define objectives, plan team composition, and final mobilization.  “No-regrets” and longlisting policy: multiple candidates prepared per role.  Strong diversity focus (personality, gender, ethnicity) in team configuration. Staff rotation supports register development and skills strengthening. | Team configuration is defined on a case-by-case basis.  15 days maximum rotation for the roster of volunteers by default. | The crisis cell assembles the first Rapid Repones Team (RRT). The team leader can then set thematic priorities based on the initial assessment results and proposed lines of action; and, if necessary, request RRT modules.  Balances senior and junior experts; generational change challenges continuity of humanitarian experience. | The National Crisis Management Center (NCMC) with NEMS and LEMT management team selects the most suitable candidates for all team functions. | The selection of the team members from the pool of available staff is done by the Team leader after consulting with: DES on obtaining a situation update on the disaster, the Head of the hospital for the medical staff, and the GIES representative for selection of logistical staff. |
| **Assessment team deployment** | ~8 members: team leader, logistician, liaison officer, medical professionals, safety & security coordinator Minimum: team leader and medical doctor. | The assessment team includes a national and regional civil protection officer, an experienced logistician, and an experienced healthcare provider. | Minimum: Team Leader and Senior Medical Officer. |  | 2-person team (where feasible) | Small initial RRT of 3 specialists |  |  |
| **3.4 Logistics** | | | | | | | | |
| **Logistical partners** | DEMA & the Danish Defense Joint Movement and Transportation Organization (JMTO)  DEMA and JMTO have a stand-by agreement that ensures a fast response 24/7. | Regional Mobile Emergency Response Unit  Regional health care system support agency (administrative, technical, and logistical)  The regional Civil Protection column including the regional Red Cross Committee and similar national organizations called Pubblica Assistenza and Misericordie | The Directorate of Civil Protection  Global commercial logistics partner Schenker |  | Ministry of Defense (provides hangar for storage of EMT in Madrid) | Swiss Armed Forces  RRT ADRIANA | Health Emergency Situation Center (HESC) | The regional Inspectorate for Emergency Situations of Bucharest and Ilfov Region (ISUBIF).  National Crisis Management Centre |
| **Pharmacy and medical equipment storage** | DEMA has a stock of medicine and medical consumables for one week of operations stored to ensure rapid deployment and has an agreement with a national pharmacy regarding the delivery of all necessary medication for deployment within 6-12 hours from receiving a request.  DEMA can procure 30 portions of blood from a hospital. | The Pisa University Hospital stores drugs, medical aids, and electro-medical equipment. Drugs are replaced before expiration and integrated into the hospital's supply flow where possible.  Electromedical equipment that needs continuous operation (e.g., laboratory equipment) is stored at the hospital and is made readily available in case of departure.  To ensure 14 days of self-sufficiency, a pre-arranged batch of drugs and prescriptions is to be delivered by the regional logistical support agency. | Medications will be stored at the Southern and Eastern Norway Pharmaceutical Trust and Norwegian Institute of Public Health.  The medications will be in addition to the hospital stock and therefore be rotated on a regular basis. |  | The equipment and materials needed for the deployment are positioned in a hangar, property of the Ministry of Defense, whose staff at the base also assist AECID by providing logistical support for loading aircraft.  Medicines are stored separately from the other materials at the contractor’s premises. |  | Medical equipment and medication are stored in a warehouse near Vilnius airport. Health Emergency Situation Center is responsible for maintaining the operational readiness of the medical equipment. | Pre-arranged batches of medical materials and drugs are handled through the Emergency Department of the Bucharest Emergency Clinical Hospital as part of daily responsibilities of the pharmacy and soon-to-expire drugs are rotated. |
| **3.5 Financial mechanisms** | | | | | | | | |
| **Funding responsibility** | Ministry of Defense (MoD) and MFA | National and Regional Department of Civil Protection and the NGO Emergency Surgery Group | Funding for the management of the EMT is shared responsibility of the MoH and the MoJ.  Deployment funding depends on the requester, which may include the MoH, MoJ, MFA, or another requesting entity. | FCDO | AECID | SDC | NCMC – Government of Lithuania - MoH | GIES |
| **Funding sources, access and conditions** | The EMT can request funding from the MoD and the MFA.  Two funding pools managed by the MoD and MFA are available each with $700,000 available.  DEMA can utilize approximately 100.000 USD for mission deployment without prior approval from the MFA (no-regret policy). | Financial coverage in the mission is provided by the Emergency Surgery Group for own funds received from donations and fees and regional funds.  The Department of Civil Protection provides ad hoc funds that are renewable during the mission for proven needs.  The National and Regional Department of Civil Protection ensure the financial coverage of activities according to the law and the specific emergency rules in place. | The MFA funding approval is based on policies underlying the humanitarian budget.  In cases of humanitarian crisis in countries not approved by recipients of assistance under the OECD DAC regulations, the MoJ and MoH will jointly consider alternative funding options. | UKMed receives core program funding as well as mission-specific grants through the FCDO.  Since the restructuring of the UK Emergency Deployments Team program, a contingency budget was introduced, which enables faster decision-making and increased flexibility for small- and medium-scale missions. | Funding comes from AECID’s budget covering staff salaries, transportation, accommodation and food costs associated with the mission.  AECID states that all necessary arrangements have been made to finance any cost related to deployment and operation. | Funding is covered by government budgets. The Federal Council drafts the International Cooperation Strategy, including proposed funding, and submits it to Parliament for approval. |  | GIES is responsible for all funding from Romania state budget, and yearly, an agreed amount of finance is directed.  Funding related to the cost of drugs and medical materials, is covered by the Bucharest Clinical Emergency Hospital.  Upon request, rapid financial support can be provided by the SMURD foundation through a strong partnership. |
| **EMT budget estimations** | Approximate deployment costs 20.7 million DKK (3 weeks).  Salaries: 7.4 million DKK, with an additional 2.3 million DKK for costs such as vaccinations, visas, uniforms, and accommodation. Mission-specific costs are transport, equipment, consumables, local staff salaries, and post-deployment refurbishment, almost 11 million DKK. The advance team budget estimate is 550,000 DKK.  Some costs are described to be eligible for EU Civil Protection Mechanism for co-financing (covering up to 75%). |  |  | The FCDO contributed  £4.68 million in 2023 and £7.14 million in 2024 to UKMed for program funding.  As an example, the FCDO  granted additional mission-specific funds to Turkey (£1.18M in 2023; £320K in 2024) and Gaza (£2.75M in 2024). |  |  |  |  |

**Abbreviations:**

AECID – Agency for International Development Cooperation

DAC – Development Assistance Committee

DEMA – Danish Emergency Management Agency

DES – Department of Emergency Situations

DOH – Directorate of Health

EMT – Emergency Medical Team

EU – European Union

FCDO – Foreign, Commonwealth & Development Office

GIES – General Inspectorate for Emergency Situations

HESC – Health Emergency Situation Centre

HQ – Headquarters

HR – Human Resources

ISUBIF – Inspectorate for Emergency Situations of Bucharest and Ilfov Region

JMTO – Joint Movement and Transportation Organization

LEMT – Lithuanian Emergency Medical Team

MFA – Ministry of Foreign Affairs

MoD – Ministry of Defense

MoH – Ministry of Health

MoJ – Ministry of Justice

NCMC – National Crisis Management Centre

NEMS – National Emergency Medical Services

NGO – Non-Governmental Organization

NHS – National Health Service

OECD – Organization for Economic Co-operation and Development

RRT – Rapid Response Team

SDC – Swiss Agency for Development Cooperation

WASH – Water, Sanitation and Hygiene
